# Barriers and facilitators of education provided during rehabilitation of people with spinal cord injuries: A qualitative description

**DOI:** 10.1371/journal.pone.0240600

**Published:** 2020-10-15

**Authors:** Alessio Conti, Valerio Dimonte, Antonella Rizzi, Marco Clari, Silvia Mozzone, Lorenza Garrino, Sara Campagna, Alberto Borraccino

**Affiliations:** 1 Department of Public Health and Pediatrics, University of Torino, Torino, Italy; 2 Spinal Unit, A.O.U. Città della Salute e della Scienza Hospital of Torino, Torino, Italy; Fordham University, UNITED STATES

## Abstract

**Background:**

After a spinal cord injury (SCI), individuals must acquire their maximum level of independence before returning to their previous social and working conditions. The education provided during rehabilitation is one of the basic but complex aspects that influence the health perspectives of people with SCI. Gaining the perspective of SCI survivors experienced barriers and resources to enhance the education process may assist healthcare professionals in understanding this complex aspect of their practice. Through a qualitative descriptive analysis, this study aimed to identify the perceived barriers and facilitators of education provided during the rehabilitation of individuals with SCI.

**Methods:**

A purposive sample of 22 adults with SCI and at least six months of home experience was recruited. Participants were assigned into four mini focus groups according to their level of independence. The focus groups were audio-recorded, transcribed verbatim, and analysed using a thematic analysis.

**Results:**

Three themes were identified: the readiness to education, the individual characteristics, and the environmental and social characteristics influencing education. Participants perceived education to be an ongoing process made up of consecutive phases, each of which had to be overcome before participants felt ready to reappraise their health and well-being. This process was affected by individual, environmental, and social factors.

**Conclusions:**

Education is constantly provided by all members of the rehabilitation team. These must stress the relevance of the contents presented, increase SCI survivors’ motivation to set achievable goals, and consider filling the gap that the patients perceive between rehabilitation centres and available community resources. The findings of this study promote the design of structured educational programmes, increasing knowledge, and improve the health perspective of SCI survivors, their families, and providers.

## Introduction

Spinal cord injury (SCI) is one of the most complex, debilitating health conditions a human being can suffer. More than 500 000 people worldwide suffer a SCI annually, with severe consequences for the families of affected individuals and on many activities of daily living (ADL) [1]. The subsequent, and sudden loss of motor-sensory and autonomic neurologic functions [2] can lead to impairments and limitations in domestic, employment, and leisure activities, causing difficulties in the maintenance of and participation in social relationships [3]. These limitations can reduce independence, affect long-term health and quality of life (QoL), increase the risk of secondary health conditions, and increase the SCI mortality rate compared to the general population [4, 5]. Moreover, challenges experienced by people with SCI, included those related to their lack of energy due to their condition, might lead to adverse psychological outcomes, such as depression and loss of motivation [6]. All these factors have direct and indirect effects on overall healthcare costs.

Rehabilitation following SCI is long and usually takes place in a dedicated rehabilitation centre. These facilities are staffed with healthcare professionals from different disciplines, who are responsible for helping those with SCI reacquire their independence and assume responsibility for their health [7]. In fact, common issues that people with SCI experience when returning to their previous social and working conditions are limited mobility, continence care, and sexuality. One of the main components of SCI rehabilitation is education. Educational programmes are designed to teach patients to master the self-management skills needed to perform ADL [8]. Appropriate self-management involves a wide array of behaviours [9] aimed at improving QoL and reducing secondary health conditions, re-hospitalisation rates, and overall SCI mortality [10].

Although the discharge from the rehabilitation centre is followed by a critical phase, when individuals must adapt to the challenges of living with their disability [11], people with SCI are often discharged before they have mastered the necessary self-management skills. This due to the progressive decline in length of stay in rehabilitation centres, shortage of staff, and skill-mix related issues [12]. Thus, practical, and cost-effective strategies that can be implemented in educational programmes early in the rehabilitation process need to be identified [13]. Recent studies showed conflicting evidence on the effectiveness of available educational programmes in improving patients’ problem-solving skills, their knowledge of the injury, self-management skills, and QoL [14, 15]. Several clinical and environmental factors have been associated with successful educational programmes in early rehabilitation; most are related to the perspectives that those with SCI have towards health, their access to such programmes and their engagement in the rehabilitation process [15]. In light of this, we must deepen our understanding of the educational experiences of individuals during the rehabilitation process to identify perceived barriers and facilitators of educational programmes. We must also identify additional resources that individuals with SCI perceive as useful to promote self-management skills and increase well-being. Hence, we aimed to identify the perceived barriers and facilitators of education during rehabilitation of individuals with SCI.

## Materials and methods

### Study design and participants

A qualitative descriptive design using mini focus groups (FGs) was chosen for this study [16]. A purposive sample of SCI survivors hospitalised and rehabilitated at the “Città della Salute e della Scienza” Hospital of Turin was selected from the hospital’s database to consider individuals who followed the same educational programme. To be eligible, patients had to: (i) have a diagnosis of SCI according to the international standard for neurological and functional classification (ASIA), (ii) be 18 years or older, (iii) have been discharged from the rehabilitation centre from 6 months to 5 years before the beginning of the study, and (iv) speak and understand Italian. Nursing home inpatients and people with cognitive impairments were excluded. Ethical approval from the Ethics Committee of the Città della Salute e della Scienza di Torino, Mauriziano Hospital, ASL TO 1, Turin, Italy (Resolution n° 105785/2016—#CS2/28), was obtained.

Thirty-one eligible patients were identified and contacted by phone, invited to participate, and asked to report their level of independence in ADL. In order to create groups based on similar individual experiences [17], patients were assigned to FGs according to their self-reported level of independence: totally dependent (FG 1), partially dependent (FG 2), fully independent (FG 3), and ambulatory and fully independent (FG 4). Although all 31 patients agreed to participate in the study, nine were unable to take part for personal reasons. A final sample of 22 participants (Table 1) attended the FGs and were included in the analysis: 15 men and six women, mainly with paraplegia (n = 14; 64%), with a mean age of 49 years (±15.4) and an average of 4.5 years (±1.7) since discharge from the rehabilitation centre. The majority of the sample was in a relationship (n = 14; 64%) and had a high school education (n = 16; 73%). Eight participants had an active social role (37%), while the majority of the remaining were unemployed (n = 6; 27%) or retired (n = 8; 37%). All participants were voluntary and informed about the study procedure. They provided a written informed consent before the start of each FG.

**Table 1:** Sample characteristics.

N°	Gender	Age	Injury level	Years since injury	Level of independence	Group n°
**1**	Male	62	Tetraplegia	3	Totally dependent	1
**2**	Male	60	Tetraplegia	3	Totally dependent	
**3**	Male	60	Tetraplegia	5	Totally dependent	
**4**	Male	33	Tetraplegia	5	Totally dependent	
**5**	Male	27	Tetraplegia	9	Totally dependent	
**6**	Female	58	Paraplegia	2	Partially independent	2
**7**	Female	55	Paraplegia	2	Partially independent	
**8**	Male	62	Paraplegia	3	Partially independent	
**9**	Male	55	Paraplegia	4	Partially independent	
**10**	Male	20	Tetraplegia	3	Partially independent	
**11**	Male	28	Tetraplegia	6	Partially independent	
**12**	Male	53	Paraplegia	6	Independent	3
**13**	Female	33	Paraplegia	6	Independent	
**14**	Male	52	Paraplegia	2	Independent	
**15**	Male	29	Paraplegia	4	Independent	
**16**	Female	57	Paraplegia	5	Independent	
**17**	Male	34	Tetraplegia	3	Ambulatory	4
**18**	Female	47	Paraplegia	5	Ambulatory	
**19**	Female	69	Paraplegia	5	Ambulatory	
**20**	Male	51	Paraplegia	6	Ambulatory	
**21**	Male	55	Paraplegia	6	Ambulatory	
**22**	Female	78	Paraplegia	5	Ambulatory	

### Procedures

FGs took place over 4 weeks (one FG per week) in September 2016 in a dedicated room at the “Città della Salute e della Scienza” Hospital. To avoid any influence on participants’ discussion, a separate area of the hospital was set up to host caregivers during the FGs. All FGs were performed once and followed the same procedure: a short trigger movie was shown at the beginning to encourage discussion and a FG guide was used to lead the subsequent discussion (Table 2). Participants were free to interact using their own names if they chose to. To maintain anonymity and confidentiality during data analysis and reporting, a sequential number was assigned to each participant. An experienced moderator (AR) facilitated each FG. Two research assistants, with a background in SCI rehabilitation, were involved as silent observers to take detailed field notes [18]. All the research team did not have previous relationship with participants or were part of the hospital staff. Each FG lasted approximately 80 minutes and was audio-recorded and transcribed verbatim. Debriefings were conducted after every FG to synthesise observations and capture initial thoughts about the topics discussed.

**Table 2:** Interview guide.

Question 1	What does it mean to be fine, to all of you? What makes you feel good today, after the injury? What does it mean to feel good in your daily life?
	*Probe 1*.*1*
	*After discharge from the rehabilitation centre*, *feeling good has assumed a different meaning;*
	*Compared to what you had in the past*, *how has the concept of well-being changed*?
Question 2	How do you take care of your health? In other words, which of the behaviours you (or your caregiver) learned during hospitalisation are most important for your health?
	*Probe 2*.*1* *Which behaviours are directed to take care of your health*?
	*Probe 2*.*2* *How do you feel and how do you see your body*, *now*?
	*Probe 2*.*3* *Can you describe more in details how [the behaviour] was learned during the early rehabilitation*.
Question 3	Do you feel you recall all the actions or patterns of actions learned during the early rehabilitation to take care of your health, when you are back home?
	*Probe 3*.*1* *Do you feel the need of having any further means to recall*, *to be sure you have not forgotten or to refresh something*?
	*Probe 3*.*2* *Is there any tools or methods you believe could be of help in fostering what you acquired and has to continue at home*?
Question 4	In case of any further need of help or information, who would you preferably ask for?
	*Probe 4*.*1 Where did you find it [source of information]*?
Question 5	If you were to talk to a peer during his early rehabilitation, what would you tell him? Which suggestions would you give to help him taking care of himself?
	*Probe 5*.*1* *What are the basic skills that an individual should have learned before hospital discharging*?

### Analysis

A thematic analysis approach [19] was applied consistently with the following phases: familiarisation with the data, generation of initial codes, searching for themes, reviewing themes, defining and naming themes, and producing the report [19]. AR, AC, and SM analysed inductively the whole set of data through an iterative process. Verbatim transcriptions of FGs were read individually, thoroughly, and repeatedly to allow the researchers to familiarise themselves with data; coding was developed to create categories and abstractions. After this first step, the researchers met to compare obtained codes and arrange final categories, which were then condensed into themes through discussion. Data collection and analysis tended to be simultaneous, to inform subsequent FGs [20]. The analysis involved two other independent auditors (SC, AB), who further reviewed categories, abstractions, and themes. Data saturation, defined as information redundancy or the point at which no new themes or codes emerge from data, can be hypothetically achieved after three FGs [21]. For this reason, four FGs were conducted in this study. Credibility and dependability were ensured through the use of an audit trail, verbatim transcriptions, and member checking with a subsample of participants [22]. The whole analysis was conducted on verbatim transcriptions and meaningful quotations were translated into English. The study was carried out and reported according to the Consolidated Criteria for Reporting Qualitative Research [23].

## Results

**Fig 1:**
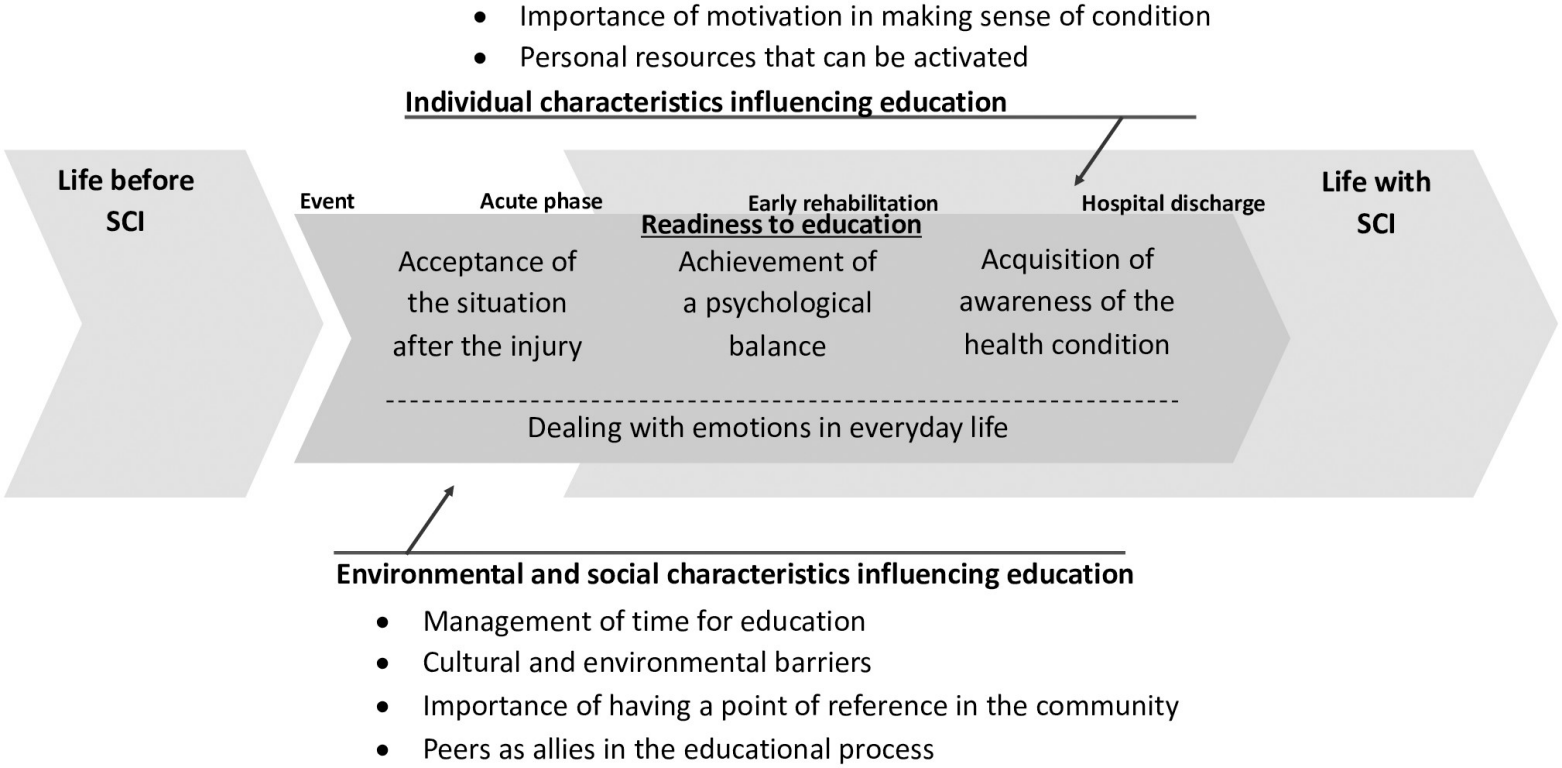
Representation of identified themes.

Three themes were identified: readiness to education, individual characteristics, and environmental and social characteristics influencing education (Fig 1). Education is an ongoing process made up of consecutive phases starting with the SCI event and then weaving through the acute, early rehabilitation, and discharge phases. In each phase, individual, environmental, and social characteristics influenced participants’ learning processes.

### Readiness to education

Hospitalisation was the first, unavoidable step on the chronological path described above, and it characterises the acute phase following the triggering event. Participants portrayed this as a phase of utter confusion and distress, when they were overwhelmed by their circumstances and unprepared to receive, let alone understand, information about their condition, regardless of how relevant this information might have been. Participants confirmed that throughout early rehabilitation, they received valuable information, but they were not able to perceive its value at the time. During the acute phase, they dealt with severe emotional distress and lack of willpower, which were amplified by the presence of pain and the lack of physical strength.

I remember that at the beginning, in my catatonic state, I felt really stuck, I couldn’t do anything. Then I learned some mechanisms [of self-management], I tested different strategies [of self-management], and I came to understand that what matters is that I still have a brain and I can still think.

(p#12–FG3)

The achievement of a psychological balance has been described as a fundamental prerequisite to engage in any educational programme, and participants could not arrive at this balance without accepting that SCI introduced a permanent change in their life. A life that, as they expressed, needed to be completely redefined. Indeed, participants recalled how their realisation and acceptance that they would not achieve total body recovery was slow and progressive, although necessary to move to the next phase of a new self-awareness and body recognition.

The problem with people who remain in the wheelchair is that they go on with their life thinking that they can’t do anything anymore. In the beginning, it happened to all of us; then we managed to get up [from the wheelchair] and we saw some light at the end of the tunnel again, but …in the beginning, if you can’t get up and you’re depressed and in a wheelchair, you don’t know if recovery is possible, because the doctors can’t tell you (…) Then, you have to decide to learn how to use the wheelchair.

(p#21–FG4)

Beyond the awareness of their new, difficult situation, participants reported feeling continuous pressure to learn numerous information and skills, in addition to following a wide array of all-important recommendations. This pressure negatively impacted their already low energy reserves which represented a further barrier to education and their ability to understand their doctors’ advice and priorities. Participants agreed that clear goal-planning during rehabilitation is as a resource to help deal with the effort required to prioritise and apply the information acquired. Moreover, they agreed that the information they were provided with during rehabilitation could be fully understood only after they had spent time at home.

You can’t expect that after a year of rehabilitation you will leave [the rehabilitation centre] knowing everything. You may leave knowing a lot of things, but you still won’t have attained all the goals you set for yourself. I have been back home for 3 years, and I still discover new things, some movements; in my opinion, our body is made to recover in its own time.

(p#19–FG4)

Participants often described their learning process as extremely slow and emotionally burdensome due to the absence of any near-term points of reference. Every single day, they faced uncertainty, never knowing what actions might represent progress and relying on trial-and-error to accomplish given goals. Moreover, the fear of failing in self-management or harming themselves led some participants to abstain from specific behaviours, relying entirely on others for the fulfilment of basic personal needs. Several participants expressed guilt for not being independent and having to rely on someone else.

It really destabilises you, (…) you are afraid that you are becoming a child again and starting from scratch, at an age that, at least in my case, is already quite advanced, isn’t it?!"

(p#14–FG3)

### Individual characteristics influencing education

Participants said that education was essential to achieve progress in the rehabilitation process. Participants’ experiences converged in the description of education as essential both in the acute phase and at home after discharge, and individual characteristics played an essential role in education. In particular, having strong motivation emerged as a crucial factor in following all provided recommendations. Participants placed great emphasis on the importance of having a personal commitment to overcoming their new condition, and to reinvent themselves in different contexts, in which working, being independent, and being useful were depicted as primary goals.

I went back to my place of work …to the city surveyor’s office, and I stayed on there, working at my drafting table until I retired. Nobody at the spinal unit could believe it. [They] came to see where I worked, what I did, how I handled it.

(p#1–FGI1)

Participants also often used the reinforcing effect of gratification to acquire the knowledge and specific skills they needed to manage their condition, which sometimes led to a tendency to put themselves above all else. At the same time, it was not easy for individuals with SCI to understand how much they could achieve using the self-learning approach, and when and to whom they could refer to get additional help when needed.

I’ve always taken poor care of myself in general. I’ve always been, I shouldn’t say it myself, but generous I am enough towards others and neglected myself a lot. (…) after the accident, I understood I had to put myself first and everything else had to come afterwards.

(p#17–FG3)

### Environmental and social characteristics influencing education

Environmental and social characteristics also appeared to have an effect on education during rehabilitation. In particular, the time needed to learn self-management skills was perceived to be excessively long in relation to the complexity of the situation, and participants agreed that they needed more time to be able to adhere to healthcare professionals’ requests. Participants expressed a general complaint towards healthcare professionals who were inflexible and presented information too quickly. They felt that professionals’ time management was mainly based on organisational needs and bureaucracy, rather than patients’ needs, creating additional distress and disorientation. Participants suggested that a possible solution for professionals should be explaining the priority given to certain activities or educational elements during rehabilitation.

They told me to take driving lessons, but I didn’t care about driving anymore, it was the last thing on my mind. I protested, because I preferred to first be independent in washing my face, dressing, not driving.

(p#11–FG2)

After discharge from hospital, participants reported that they were overwhelmed with doubts about how to apply the skills they learned during the rehabilitation process in their real lives, and they wondered if they had really acquired all the necessary information. They reported a lack of continuity between the rehabilitation centre and the community care centres and general practitioners, which were both perceived as less useful. Indeed, participants believed these institutions lacked adequate knowledge on SCI and that the rehabilitation centre was the only place they could to turn to in case of need.

When I have to come here [to the rehabilitation centre], I feel like I’m going to heaven (…) I still come here for physiotherapy, I come to use the pool, and it’s a good thing for me, it’s a fabulous thing to come here.

(p#21–FG4)

Family and loved ones were often depicted as personal resources that participants could count on to overcome the difficulties in their rehabilitation path and as the bridge that connected them with the real world, even when the price was intrusiveness. In the absence of a supporting family, participants reported that it was challenging to have complex needs fulfilled, and they described taking specific self-management information from the Internet or other alternative channels.

Fortunately, my family has kept me busy since the beginning. Really, I did not have time to think, and, in the end, it was a positive thing.

(p#9–FG2)

Participants reported that peers were another important resource in overcoming struggles and avoiding recurring negative thoughts. During the FGs, participants mentioned that sharing practical insights and facing difficulties with peers represented an important opportunity. They expressed their desire to be involved in more group activities or sports, even in early rehabilitation, when their schedule was managed by the healthcare professionals. Participants identified therapeutic trips, leisure activities, and home visits with healthcare professionals and peers as the most effective educational tools they had during early rehabilitation, as those moments were similar to their discharge experience.

Outside is different. Because reality is outside, during rehabilitation you have home visits …I got to have my first one after 4 months (…). I spent the night in my own home for the first time in October. I think that home visits are really necessary, because they make you do the things you need to do …take the car, go outside, so you can see how you can manage yourself at home.

(p#2–FG1)

## Discussion

This study allowed us to explore specific, perceived barriers and facilitators of educational programmes offered during rehabilitation among people with SCI. Participants stressed that learning was an ongoing process, made up of consecutive phases, that started during rehabilitation and continued once they were back home. Moreover, they highlighted some systemic weaknesses of rehabilitation, such as healthcare professionals’ time management, their lack of communication, and their focus on clinical outcomes. Although the need of SCI survivors for an educational follow-up, participants found a poor connection between rehabilitation and community. The awareness by healthcare professionals of the complex trajectory followed by SCI survivors, the importance of strengthening individual resources, and the involvement of peers and families may constitute resources to improve education in this population.

Consistent with other studies, rehabilitation following SCI was perceived as a phase in which every single activity, combined with medical management, is exhausting and has an effect on individuals’ readiness to learn [15]. Physical and psychological stressors experienced by SCI survivors during early rehabilitation represent barriers to their participation in educational programmes [13]. The acute phase was mainly characterised by high distress due to the injury, that is not connected to the extent of SCI but can negatively impact the coping process and the acceptance of the condition [7, 24]. Moreover, participants confirmed that the exposition to distress due to overwhelming physical changes led to difficulties understanding the relevance of educational information [8]. Our findings confirmed that, following an acquired disability, people experience a deep sense of grief [25], that in SCI has been described as a body/mind separation, in which one’s own body is perceived as a dead person [26]. Longitudinal studies showed that grief-induced responses occur immediately after a loss and decline within the following 6–12 months, when pre-loss functions are recovered [27, 28]. Within this period, participants reported they faced difficulties in understanding, remembering, and applying educational information and recommendations. The perceived decline in grief lines up with the moment of acceptance [29], thus allowing a balance between the maintenance of pre-injury goals and the adaptation to current needs [30, 31]. Therefore, considering that attempts to educate people with SCI might often fail whilst dealing with their distress, healthcare professionals should consider the assessment of individuals’ readiness to learn as an important resource to improve the effectiveness of education they provide during rehabilitation [7].

After a SCI, a large volume of information has to be acquired in a limited amount of time [32]. This generates a sense of frustration, as participants are forced to learn several skills quickly while they are distressed, and is difficult to understand the link between the information provided and its broader scope [13] because they have not yet experienced ADL and related secondary conditions [33]. It follows that the length of stay in inpatient rehabilitation was perceived to be insufficient when considering the sheer volume of information that had to be acquired [12]. Consistently with other studies, healthcare professionals were often seen as inflexible, with learning priorities that seemed to be different from those set by participants [34], who reported feeling that they were discharged before their educational needs were met. As a result, participants felt they could only rely on a trial-and-error approach, which has already been reported to happen when expert guidance is lacking [35]. Part of these behaviours in professionals could be explained by the scheduling issues and resource scarcity affecting educational programmes [32]. However, healthcare professionals should select the educational topics and required skills according to the various stages of rehabilitation, their perspectives, and those of people with SCI [11, 13]. Moreover, it is fundamental to structure educational programmes justifying the recommendations provided, and their link with everyday life. Real-life scenarios, problem-based learning, e-learning, or even virtual reality are promising tools that can be applied in educational programmes to foster self-management skills and encourage self-direction [32]. Our findings further encourage the implementation of specific training for healthcare professionals to improve patient-clinician communication, which could help staff better understand patients’ needs and expectations. This can help in setting tailored, achievable goals, strengthening the partnership between patients and rehabilitation staff, and progressively humanising the learning process after SCI [34].

Participants emphasised the difficulties experienced by individuals with SCI in metaphorically “cutting the cord” from the rehabilitation centre, where environmental, cultural, and economic barriers do not exist [36, 37]. Individual characteristics, such as hope and motivation, are crucial to foster the connection between new life perspectives and past interests, and should be promoted throughout the rehabilitation process towards a psychological support [8]. Interventional approaches, including problem-focused activities and cognitive behavioural therapies, were suggested to be effective in promoting self-acceptance and post-traumatic growth, and in favouring positive adjustment and maintenance after rehabilitation [38]. At discharge, the presence of environmental and social barriers strengthened the role of the family and peers in overcoming difficulties. Family caregivers have received increasing attention for their fundamental responsibilities in maintaining SCI survivors’ motivation and well-being [39]. Moreover, peer support, especially during sports and leisure activities, has been shown to help people with disabilities feel socially recognised, as well as enhancing education effectiveness [40]. Involving families and peers to overcome distress in the acute phase, even in recreational sports or group activities, was found to be helpful for people with SCI, as it improved self-confidence, self-efficacy, readiness to face ADL [40], and re-hospitalisation rates [34, 41].

Given the increase in life expectancy, combined with the growing number of individuals with SCI struggling to live independently in their households [33], more resources must be allocated to reduce the distance, also experienced by participants, between rehabilitation centres and the community. Community-based programmes would be suitable to bridge the gap between the rehabilitation centre and SCI survivors’ homes, but there are currently few healthcare professionals prepared to deliver such programmes [33]. A possible solution would be to encourage outpatients and community groups to develop education programmes in the community, supervised by experts from rehabilitation centres and using high tech resources to simplify communication [42]. In this connection, during the transition between rehabilitation and social reintegration before discharge, individuals with SCI need to learn about the available educational resources in their community [32]. Despite general concerns about their quality, online resources are an indispensable resource for people with SCI, as they are accessible and easy to use, gaining recently more popularity, as they can meet individual learning needs and styles [42]. Moreover, technology and telehealth is becoming popular as it enables individuals with SCI the possibility of limiting their travel and healthcare professionals to monitor their condition [43]. In this connection, adapted technology may potentially represent an educational tool combining the possibility of regular goal setting and follow-up in this population. Future studies are needed to understand the effectiveness of educational programmes in people with SCI in terms of well-being and the occurrence of secondary conditions following discharge in order to develop programmes focused on a core set of basic skills to be provided at the correct stage of rehabilitation. In this regard, the use of standardised tools, including measures of depression or motivation, could be useful in assessing the impact of the education provided on the mental health of people with SCI.

### Limitations

The results of this study should be interpreted within its limitations, including the relatively limited sample size and the sampling procedures. Participants were chosen from the leading rehabilitation centre in the north of Italy, which has long-standing expertise in the diagnosis and treatment of SCI. The inclusion of 31 participants divided into four FGs should have guaranteed sufficient coverage of the set of concerns under investigation [21]. The main reason for refusal to participate was logistical difficulties not directly connected to the study purposes; therefore, sampling bias should have minimal effects on the study results. Even if our participants were from a specific geographical context, our findings were consistent with available studies conducted in different settings. Lastly, although researchers were external to the hospital staff, the location of the FGs could have represented a further bias, as participants may not have felt they could freely express their thoughts and still protect the professionals they dealt with during rehabilitation. This study followed a qualitative design that was valuable in describing the experiences of the participants, although it did not allow the evaluation of the influence of their clinical or socio-economic characteristics on perceived education. Effects of variables such as gender, age, injury severity, and financial status or access to services on education need to be enriched through quantitative studies that might corroborate our findings. Furthermore, as we did not have any data that could link the education provided with hospital re-admissions, our ability to determine the real effect of the educational programme on this outcome was limited.

## Conclusion

Education is one of the basic, but complex aspects of rehabilitation after SCI. Healthcare professionals should be aware of the great efforts SCI survivors must make to master this complexity, given the emotional distress perceived, and be ready to learn. Therapeutic and educational processes need to be conducted in parallel and an educational focus on a core set of information to be provided with each stage of rehabilitation is recommended. Health providers must stress the relevance of the education provided, especially when individuals with SCI and their caregivers lack experience in a home environment. Moreover, the involvement of families, peers, and community services in learning interventions during rehabilitation is highly recommended to promote the effectiveness of education and improve the well-being of SCI survivors. The development of structured educational programmes based on the experiences of healthcare professionals and people living with SCI is essential to increase knowledge and improve the health perspective of SCI survivors, their families, and providers.
